# CRISPR Comparison Toolkit: Rapid Identification, Visualization, and Analysis of CRISPR Array Diversity

**DOI:** 10.1089/crispr.2022.0080

**Published:** 2023-08-14

**Authors:** Alan J. Collins, Rachel J. Whitaker

**Affiliations:** ^1^ Carl R. Woese Institute for Genomic Biology, University of Illinois Urbana-Champaign, Urbana, Illinois, USA and University of Illinois Urbana-Champaign, Urbana, Illinois, USA.; ^2^ Department of Microbiology, University of Illinois Urbana-Champaign, Urbana, Illinois, USA.

## Abstract

CRISPR-Cas systems provide immunity against mobile genetic elements (MGEs) through sequence-specific targeting by spacer sequences encoded in CRISPR arrays. Spacers are highly variable between microbial strains and can be acquired rapidly, making them well suited for use in strain typing of closely related organisms. However, no tools are currently available to automate the process of reconstructing strain histories using CRISPR spacers. We therefore developed the CRISPR Comparison Toolkit (CCTK) to enable analyses of array relationships. The CCTK includes tools to identify arrays, analyze relationships between arrays using CRISPRdiff and CRISPRtree, and predict targets of spacers. CRISPRdiff visualizes arrays and highlights the similarities between them. CRISPRtree infers a phylogenetic tree from array relationships and presents a hypothesis of the evolutionary history of the arrays. The CCTK unifies several CRISPR analysis tools into a single command line application, including the first tool to infer phylogenies from array relationships.

## Introduction

CRISPR-Cas (clustered regularly interspersed short palindromic repeats; CRISPR-associated proteins) is an adaptive immune system present in most archaea and many bacteria that provides immunity against mobile genetic elements (MGEs) such as viruses.^1,2^ CRISPR-Cas immunity is acquired through the incorporation of spacers (small fragments of DNA from invading MGEs) into a region called a CRISPR array. CRISPR arrays are dynamic as they can both acquire and lose spacers. Over time, these events alter the length and spacer content of arrays.^3^ Spacer acquisition typically occurs at the leader end.^2^ However, under some circumstances, ectopic spacer acquisition can occur.^4,5^ Repeats located at the leader-distal end of the array (the trailer end) are thought to be older and sometimes contain polymorphisms.^6^

Because proper CRISPR function requires the recognition of specific sequence and secondary structure in the repeats,^7^ mutations within trailer-end repeats can result in the loss of function of spacers flanked by the degraded repeats. Spacers can also be lost by deletion events.^8,9^ Specifically, homologous recombination between repeat sequences can lead to the loss of a short stretch of spacers and repeats.^3,10,11^

Because thousands of unique spacers can be derived from a single virus,^12,13^ there is an enormous number of possible spacer sequences. The presence of two identical spacers in different CRISPR arrays is unlikely to have arisen by chance and may indicate that the arrays share a common ancestor (i.e., the arrays are homologous). Therefore, comparisons of spacers between CRISPR arrays can reveal phylogenetic relationships between them.

To infer phylogenetic relationships between CRISPR arrays, one must first visualize and compare the arrays with one another. The previously published tools CRISPRviz and CRISPRStudio show which spacers are shared or distinct between CRISPR arrays^14,15^; however, no tools have been developed to infer phylogenetic relationships between arrays.

In this study, we present the CRISPR comparison toolkit (CCTK), a command-line software toolkit for the analysis of relationships between CRISPR arrays. CCTK includes tools to identify CRISPR arrays and predict protospacers. CCTK also includes two new programs for the analysis of array relationships: CRISPRdiff, which visualizes arrays and highlights regions of similarity, and CRISPRtree, which infers the phylogenetic relationships between a set of arrays using a maximum parsimony approach. By unifying the above tools into a single command-line application, CCTK provides resources for identifying relationships between CRISPR arrays in a few simple steps. Furthermore, CCTK uses simple file formats to facilitate the integration of CCTK tools into existing pipelines.

## Methods

### Data set description

To illustrate the functionality of CCTK tools, we selected a previously published set of *Pseudomonas aeruginosa* isolates that were collected from a population of cystic fibrosis patients over the course of several years.^16,17^ These isolates represent repeated sampling of a number of strains circulating among patients and therefore provide a record of the evolution of those strains. Furthermore, changes in the CRISPR arrays encoded by these strains were observed over the course of the study (arrays gained or lost spacers). In addition, horizontal exchange of CRISPR arrays between *P. aeruginosa* strains may have occurred.

**FIG. 1.**
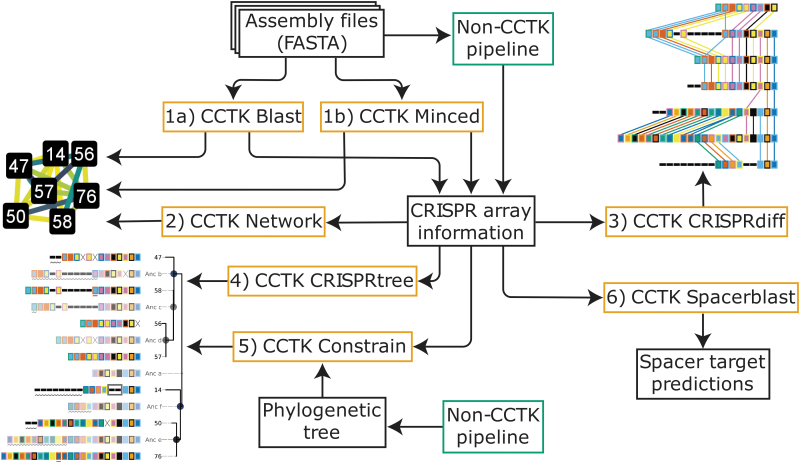
Flow of data through CCTK tools. Beginning with genome assemblies in FASTA format, CCTK tools can be used to identify and analyze CRISPR arrays. The six CCTK tools are as follows: (1a) Blast, (1b) Minced, (2) Network, (3) CRISPRdiff, (4) CRISPRtree, (5) Constrain, and (6) Spacerblast. The functions of each CCTK tool are described as follows: (1) CRISPR arrays can be identified in assemblies using either MinCED or BLASTn followed by processing steps that produce aggregated CRISPR information for all input assemblies. These CRISPR information files are used by downstream CCTK tools. Alternatively, non-CCTK tools can be used to identify arrays, and their output easily adapted to the simple file formats used by CCTK. (2) The relationships between CRISPR arrays can be represented as a network in which each array is represented as a node. Homologous arrays (i.e., arrays containing identical spacers) are connected by an edge, the weight of which corresponds to the Jaccard similarity between the two arrays. (3) Groups of homologous arrays can be visualized using CRISPRdiff to identify the differences and similarities between arrays. (4) CRISPRtree can infer a phylogenetic tree representing the relationships between homologous arrays and can hypothesize events that occurred during their history. (5) Constrain can visually represent how CRISPR arrays may have evolved given a fixed tree topology to allow the reconciliation of CRISPR relationships with other phylogenetic data. (6) Spacerblast predicts protospacer targets of CRISPR spacers and determines the presence of a PAM. CCTK, CRISPR comparison toolkit; PAM, protospacer adjacent motif.

We selected this data set to illustrate the functionality of CCTK for the following reasons. First, by collecting many samples from a single clinic over several years, mutations and changes in CRISPR arrays were recorded with high resolution. Previous analysis of this data set indicates that there are several groups of related arrays that underwent spacer gain and loss over the course of the study. Second, *P. aeruginosa* strains encode CRISPR arrays that typically contain a small number of spacers; visualizing these arrays is therefore straightforward. Finally, there is evidence of the horizontal gene transfer (HGT) of CRISPR arrays. As CCTK is designed to facilitate the exploration of related CRISPR arrays, this data set provides a simple illustrative example of CCTK functionality. The flow of information between CCTK tools is shown in Figure 1. The sequence records analyzed here and the CRISPR arrays identified are described in Supplementary Table S1.

### Sequence assembly and core genome identification

The sequence reads associated with the 72 “clone-corrected” isolates described by England et al.,^16^ first published by Marvig et al.,^17^ were retrieved from the European Nucleotide Archive. Assemblies were produced using Spades with the “careful” option.^18^ An alignment of the core genome sequences of these assemblies was identified using Spine, Nucmer, and a custom script (Supplementary Methods).^19,20^ IQTREE2 was used to infer a maximum likelihood tree from this core genome alignment (Supplementary Fig. S1).^21^

### CRISPR array identification

CCTK includes two tools to identify CRISPR arrays in genome assemblies, CCTK Minced and CCTK Blast. CCTK Minced uses MinCED, which identifies CRISPR arrays using a sliding window search to identify regularly spaced repeats and can identify CRISPR arrays without requiring the user to have prior knowledge of the expected CRISPR subtypes.^22^ CCTK Blast uses BLASTN to identify CRISPR arrays by searching for a user-defined set of CRISPR repeat sequences.^23^ Both tools can use repeat orientation information (provided by the user) to correctly orient the identified arrays.

Optionally, both tools can assess the sequence similarity between spacers to collapse groups of spacers into a single representative (when they differ by fewer than a threshold of bases specified by the user). Alternatively, CRISPR arrays identified using other approaches can be converted into the simple file format used by CCTK tools. Additional details about CCTK Blast and CCTK Minced are provided in the Supplementary Methods.

### CCTK network representation of homologous CRISPR arrays

**FIG. 2.**
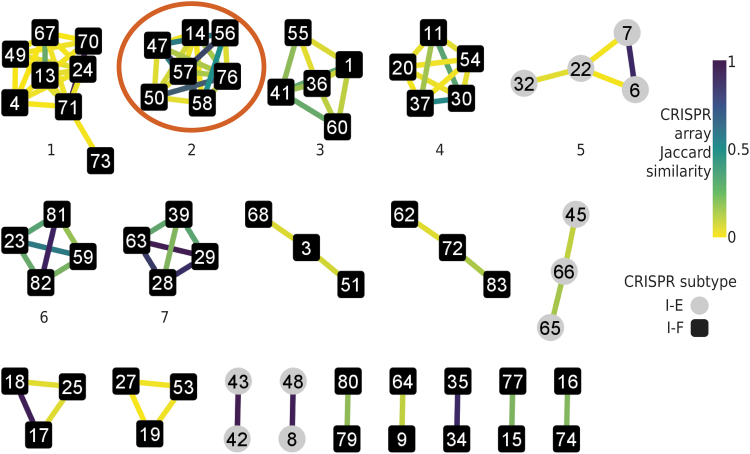
Clusters of homologous arrays can be identified using a network representation of array relationships. Network representation of spacers shared between arrays in *Pseudomonas aeruginosa* isolate clones corrected by England et al., visualized using Cytoscape. Each CRISPR array is represented by a node. An edge is drawn between two nodes when CRISPR arrays share at least two spacers. Edge color corresponds to the Jaccard similarity between the two connected arrays (i.e., the number of spacers shared between the two arrays divided by the number of total unique spacers in the two arrays) as indicated in the color key. The seven largest clusters are numbered (corresponding to the cluster numbers in Supplementary Fig. S1). Circled is a cluster, hereafter called cluster 2, which is analyzed further in subsequent figures.

CCTK produces a network in which CRISPR arrays are represented as nodes (Fig. 2). Two arrays are connected by an edge if they contain identical spacers (i.e., are homologous). CCTK quantifies homology between two arrays using Jaccard similarity: the number of spacers in common between two arrays divided by the total number of unique spacers present in the two arrays.^24^

CCTK Minced and CCTK Blast each produce a network when they are used to identify arrays. Alternatively, CCTK Network produces a network representation of CRISPR arrays identified using any method (and provided in the format required by CCTK) (Fig. 1). For all three of these tools, the user can specify the minimum number of spacers that must be shared between two arrays for an edge to be drawn between them.

### CRISPRdiff: CRISPR array visualization

**FIG. 3.**
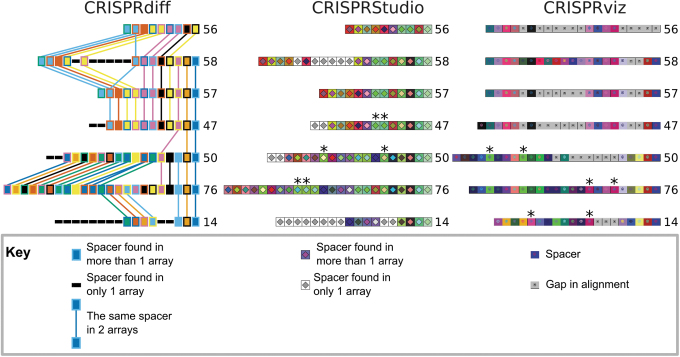
CCTK CRISPRdiff produces a clearer illustration of array relationships than the previously published tools. Cluster 2 arrays were visualized using each of the following tools: CCTK CRISPRdiff, CRISPRStudio, and CRISPRviz. The three tools all represent CRISPR spacers as colored squares and each row of colored squares represents a CRISPR array. Each spacer is assigned a unique combination of two colors; when squares with the same colors are seen in two arrays, it indicates that the spacer is present in both arrays. Below the visualization of each tool is a key describing the elements of each visualization. The leader end of each array is on the left and the trailer end is on the right. CCTK CRISPRdiff and CRISPRviz show arrays identified using MinCED, while CRISPRStudio shows arrays identified using its companion tool, CRISPRdetect. The plot produced by CRISPRStudio shows an additional spacer with 10 mismatches at the trailer end of each array that was only identified by CRISPRdetect. CRISPRStudio was run using the –gU option to assign unique spacers a gray fill color. The automatic spacer alignment function of CRISPRviz was used, followed by manual correction (gaps in the alignment are represented as gray boxes). The order of arrays in the images produced by CRISPRStudio and CRISPRviz was manually altered to correspond to the order chosen by CCTK CRISPRdiff. Array identifiers assigned by CCTK Minced are shown next to each corresponding array. “*” indicates spacers that were assigned colors with low visual contrast by CRISPRStudio or CRISPRviz.

CRISPRdiff visualizes CRISPR arrays through three steps. First, spacers that are present in more than one array assigned a unique combination of fill and outline color. Unique spacers are represented as thin black rectangles (Fig. 3). Next, the order of arrays in the plot is determined to maximize the number of spacers shared between adjacently plotted arrays. Finally, the plot is drawn using Matplotlib, which allows the plot to be saved as many common file formats.^25^

Spacers are assigned colors using either a default colorblind-friendly color scheme^26^ or user-specified colors. Array order is determined by an approach that maximizes the number of spacers in common between adjacently plotted arrays. For small numbers of arrays, the best possible order is found. For larger numbers of arrays, a limited search is performed, and the best order found is used. The user can also set the array order. See the Supplementary Methods for additional details about color assignment and array order determination.

### CRISPRtree: Maximum parsimony analysis of CRISPR array relationships

Given a list of arrays to be analyzed, CRISPRtree constructs a tree starting with two arrays and then adds the remaining arrays to the tree one by one. The process used by CRISPRtree (illustrated in Supplementary Fig. S2) is as follows. First, two CRISPR arrays are aligned using the Needleman–Wunsch algorithm (Supplementary Fig. S2B).^27^ Each spacer is treated as a character and aligned using the following scores: match: 100, mismatch: -1, gap: -2. These scores were empirically chosen to ensure that shared spacers are always aligned.

The aligned arrays are then split into modules. Modules are classified by identifying sets of consecutive spacers that have the same relationship between the aligned arrays (Supplementary Fig. S2B). For example, a set of consecutive spacers present at the leader end of one array, but missing in the other, are classified as a “leader-end acquisition module.” A set of consecutive spacers present in both arrays are classified as a “shared module.”

Next, a hypothetical ancestor array is generated by using an evolutionary model of CRISPR array change that involves the following possible events: leader-end acquisition, the loss of spacers one at a time from the trailer end, and deletion or insertion of one or more spacers anywhere in the array (Supplementary Fig. S2C). The process by which CRISPRtree processes an array alignment to infer an ancestral array is described in the Supplementary Methods.

After a hypothetical ancestral array has been inferred, events in each descendant array are identified and parsimony costs assigned. Events are identified by processing each descendant of the newly generated ancestral array as follows (Supplementary Fig. S2B). First, the descendant array and ancestor array are aligned. Then, modules of spacers are identified in the descendant array as described above (Supplementary Fig. S2B). Next, each module is associated with a type of event (i.e., acquisition, insertion, trailer loss). Finally, the parsimony costs for all identified events are summed. The total parsimony cost of events identified in a descendant array is set as the branch length between the array and its ancestor in the tree. Default event parsimony costs were empirically determined. Default event parsimony costs are acquisition: 1, duplication: 1, insertion: 30, deletion: 10, trailer loss: 1, independent acquisition: 50. These costs can also be set by the user.

Once the tree has been initialized with the first two arrays and their hypothetical ancestral array (Supplementary Fig. S2D), additional arrays are added to the tree. To add an array to the tree, it is first compared with each array already present in the tree (Supplementary Fig. S2E). The closest match already present in the tree is then set as the sibling node of the newly added array and a hypothetical ancestor is generated (Supplementary Fig. S2F–H).

When all arrays have been added to the tree, any ancestral arrays that are themselves identical to their ancestor (i.e., internal nodes with branch length of zero) are collapsed to a polytomy. Tree manipulation is performed using the python package DendroPy.^28^

Different trees are generated in this manner by adding arrays to the tree in different orders. Each tree is then scored according to the total branch length of the tree (i.e., the total parsimony cost of all hypothesized events). The tree (or trees if multiple equally parsimonious trees are found) with the lowest total parsimony cost is considered the best. Trees are output in Newick format and visualized using Matplotlib.^25^

Optionally, a measure of node support can be calculated by CRISPRtree. Node support is calculated using the replicates of the tree building process performed by CRISPRtree. First, each input array is assigned a position in a binary string. Then, every tree produced by CRISPRtree is encoded as a list of binary strings. Each element in the list represents an internal node in the tree and the binary string indicates which arrays are descendants of the node. For example, for three arrays A, B, and C (which are assigned positions in a binary string in alphabetical order), an internal node with A and C as descendants would be encoded as 101.

Once all trees are encoded in this way, the nodes in the most parsimonious tree(s) are compared with all other trees, and the number of times that each node is seen is counted. This count is then divided by the total number of trees constructed, resulting in a proportion of trees containing that node. Node support is reported in the Newick string and indicated using a number or colored circle at the corresponding internal node in the graphical representation of the tree.

### Constrain: Inference of CRISPR array evolution given a fixed tree topology

Given a phylogenetic tree, Constrain infers an evolutionary history for a set of arrays that fit the provided topology. The process used by Constrain is as follows. First, the leaves on the input tree are populated with CRISPR arrays. Then, starting with leaf nodes and working toward the root of the tree, the array at each node is aligned with the array at its sibling node and an ancestral array is hypothesized using the same approach as that used by CRISPRtree (illustrated in Supplementary Fig. S2B, C). Once all internal nodes in the tree have been populated with hypothetical ancestral arrays, each array is annotated with events that would have occurred for it to have arisen from its ancestral array.

### Tree comparisons

The arrays produced by each simulation performed using Evolve (see Supplementary Methods) were analyzed with CRISPRtree. The CRISPRtree output was compared with the true tree (produced by Evolve) using Robinson–Foulds (RF) distance, which measures the number of partitions implied by one tree but not by another.^29^ RF distance was calculated using the ete3 toolkit version 3.1.2 in python3.8.12.^30,31^

To compare the RF distance between simulation replicates (in which trees with different numbers of nodes may be produced), each distance is expressed as a fraction of the maximum possible RF distance between the two trees, indicating the extent to which the two trees differ relative to two completely distinct trees.

This analysis was performed for simulations run with different parameters: numbers of events (e.g., the acquisition of a spacer), which controls the size and extent of divergence between arrays; the rate of loss of arrays, which impacts the level of incompleteness of the data set and the extent to which extant arrays are diverged; and the relative rates of spacer acquisition and deletion, which determines the type of relationships between arrays and, at high levels of deletion, the extent to which the relationships between arrays is obscured by spacer loss. Parameters used in these simulations are described in more detail in the Supplementary Methods.

### Performance assessment

Running times are reported for CCTK tools run using default settings on a single thread. The test system used is running Ubuntu 20.04.5 in Windows Subsystem for Linux 2 in Windows 11, Intel I9-10850k, 64GB RAM.

### Software availability

All source code is available on GitHub (https://github.com/Alan-Collins/CRISPR_comparison_toolkit). CCTK can be installed either by downloading from the GitHub repository or using the package manager Anaconda (https://anaconda.org/bioconda/cctk). Documentation describing usage of CCTK tools is available at https://crispr-comparison-toolkit.readthedocs.io/en/latest/

CCTK relies on the following dependencies (version numbers in use at publication): Python3.8, DendroPy 4.5.2, Matplotlib 3.5.0, NumPy 1.21.2, MinCED 0.4.2, and BLAST +2.12.0+.^22,23,28,31,32^

## Results

### CRISPRdiff compares arrays and visualizes the relationships between them

In this study, we analyzed a data set of previously described *P. aeruginosa* isolates.^16,17^ The phylogenetic relationships between these isolates are shown in Supplementary Figure S1. In the following sections, we present analyses using CCTK to characterize the relationships between CRISPR arrays encoded by these *P. aeruginosa* isolates.

We first used CCTK Minced to identify CRISPR arrays (Supplementary Table S1) and construct a network representing the sharing of spacers between homologous arrays (Fig. 2). CCTK Minced was run with the option “-s 2” to group spacers differing by two or fewer bases. The cluster of arrays circled in Figure 2 (referred to as cluster 2 arrays) is used to illustrate the functionality of CCTK tools. Except where stated, all the subsequent analyses presented focus on cluster 2 arrays.

Visualizing array relationships as a network enables the easy recognition of sets of homologous and unrelated arrays. Comparison of the spacers that are shared between homologous arrays can then be used to further explore the relationships between those arrays. There are two previously developed tools for visualization and comparison of CRISPR arrays: CRISPRviz and CRISPRstudio.^14,15^ While both tools can be used to produce visual representations of CRISPR arrays, they have limitations when used to identify similarities among a group of arrays. Therefore, we developed a new visualization tool, CRISPRdiff, as part of CCTK, to more effectively identify the differences and similarities between arrays.

Key elements that differ between CRISPRdiff, CRISPRstudio, and CRISPRviz are described in Table 1, and the visualizations produced by the three tools are shown in Figure 3. The most significant advantages of CRISPRdiff over CRISPRstudio and CRISPRviz are discussed further here.

**Table 1.** Comparison of CRISPR array visualization tools

	CCTK CRISPRdiff	CRISPRStudio	CRISPRviz
**Distinguishing feature**	Highlights shared spacers in a small number of related CRISPR arrays	Group and visualize large number of CRISPR arrays from all input assemblies	Interactive exploration of CRISPR arrays through click and drag control of array order
**Installation**			
Source	GitHub, Anaconda	GitHub	GitHub, Docker
Admin/sudo required?	No	Yes	No
**Upstream analysis**			
Require array identification first?	Yes	Yes	No
CRISPR-identification tools	CCTK Minced, CCTK Blast, other with reformatted output	CRISPRdetect only	MinCED only
Input file	.txt array details file	.gff from CRISPRdetect	.fasta assemblies
Determine array orientation	Done by CCTK if specified by user	Done automatically by CRISPRdetect	Not possible
Collapse similar spacers	User-set mismatch threshold	User-set mismatch threshold	Fixed 2 mismatch threshold
**Control of plot elements**			
Array order control	Automatic, manual	Automatic, manual	Automatic
Array order automatic method	Maximize spacers shared between adjacent arrays	Markov clustering	File order
Output format	Any Matplotlib format (PNG, SVG, JPG etc.)	SVG	Screenshot of browser window (PNG)
Plot dimensions	Specify in command line	Resize in graphic design software	Resize in graphic design software
Which arrays are plotted	User specified	All present	All present
Postprocessing of image required	None	Remove unwanted arrays	Remove unwanted arrays, manually reverse spacer order, and reverse complement arrays to identify homologous arrays
**Identification of shared spacers**			
Indication of same spacer	Same color, joined with a line	Same color	Same color, same shape
Color scheme	Built-in, user-specified	Random	Semirandom (DNA sequence converted to numerical RGB values)
Colorblind-friendly	Yes (default color scheme)	No	No
User control of color scheme	Yes (provide color scheme or specify for each spacer)	No	No
Reproducible color scheme	Yes (optional)	Yes (optional)	Yes

CCTK, CRISPR comparison toolkit.

The ability to identify shared spacers is important when comparing homologous arrays. CRISPRdiff uses lines connecting identical spacers to clearly highlight the presence of shared spacers. In addition, CRISPRdiff uses a colorblind-friendly color palette that ensures visually distinct colors are assigned to different spacers.^26^ Alternatively, CRISPRdiff allows the users to define their own color scheme. CRISPRStudio and CRISPRviz produce visualizations that may include colors that are not visually distinct or colorblind friendly (spacers indicated by asterisks, Fig. 3; Supplementary Fig. S3; Table 1), which makes it difficult to identify shared spacers.

Unlike the other tools, no further processing of the output image is required for CRISPRdiff. Instead, which arrays are included in the plot produced by CRISPRdiff can be specified in the command line. In contrast, when using CRISPRStudio or CRISPRviz, the user must manually process the output images to remove unwanted arrays that were identified in the input assemblies. Furthermore, in the case of CRISPRviz, the user is also required to manually experiment with reverse-complement and reversing the order of spacers in arrays to visually identify homologous arrays.

### CRISPRtree infers array relationships using maximum parsimony

CRISPR arrays preserve a record of the MGEs encountered by a cell. In addition, the changes that have occurred within a CRISPR array over time represent a history of the evolution of that array. Comparing homologous CRISPR arrays and reconstructing the events that may have occurred during their evolution allows us to infer the phylogenetic relationships between CRISPR arrays and the genomes in which they are located.^6,33^ However, no tools have previously been developed to analyze the phylogenetic relationships between homologous CRISPR arrays. We therefore developed CRISPRtree, which infers the phylogenetic relationships among a set of arrays using a maximum parsimony approach (see the Methods section and Supplementary Methods; Supplementary Fig. S2).

**FIG. 4.**
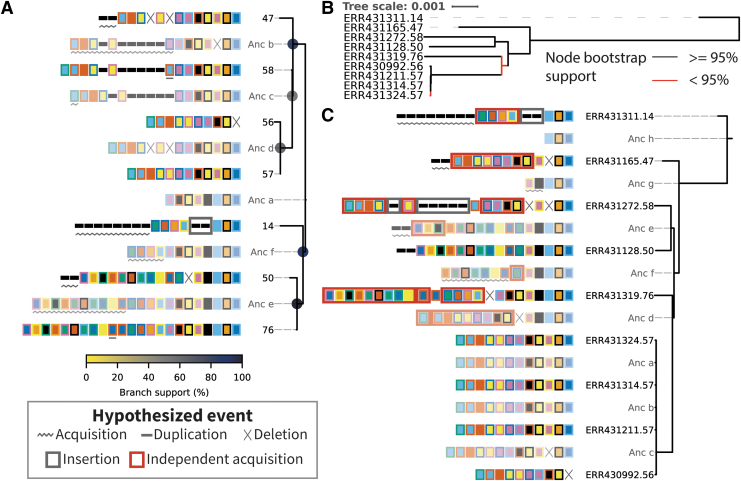
CCTK CRISPRtree can infer a phylogenetic tree from array relationships, and CCTK Constrain can assess other phylogenetic data. **(A)** CRISPRtree was used to infer a tree representing the relationships between cluster 2 arrays. Leaf nodes in the tree correspond to the seven extant arrays (i.e., the arrays provided as input to CRISPRtree). Each internal node in the tree corresponds to a hypothetical ancestral state of the descendent arrays. Hypothetical ancestors are assigned names beginning with “Anc” and are rendered partially transparent to provide visual contrast. Next to each node label is a visual representation of the corresponding CRISPR array. CRISPRtree automatically annotated each array with events that are hypothesized to have occurred since the ancestor of that array (see Key). No events are highlighted on the root node array (Anc_a) as it is the last common ancestor of all extant arrays being considered and does not have an ancestral state. Branch support is indicated using colored circles at the corresponding branch (see Key). **(B)** Maximum likelihood tree for the isolates encoding cluster 2 arrays. Core genome SNPs were identified using Spine, Nucmer, and a custom script (Supplementary Methods). IQTREE2 was then used to infer a tree using a model selected by ModelFinder (UNREST+FO+R3). Red branches indicate node support below 95% as calculated by the Ultrafast bootstrap tool included in IQTREE2. Leaf names are composed of the European Nucleotide Archive accession number and the cluster 2 array ID encoded by that isolate. **(C)** Tree produced by CCTK Constrain representing the events that CCTK Constrain automatically hypothesized would have to have occurred if cluster 2 arrays had evolved according to the core genome phylogeny shown in **B**. Events are highlighted using symbols described in the Key. Leaf labels correspond to those in **B** and indicate the assembly accession and cluster 2 array ID.

Figure 4A shows the tree inferred by CRISPRtree for cluster 2 arrays. CRISPRtree hypothesizes that all seven extant arrays descended from a last common ancestor, Anc_a, containing 8 spacers. CRISPRtree automatically annotates each array with events that CRISPRtree hypothesized occurred since its ancestor (see Key, Fig. 4). Using the events annotated on each CRISPR array in Figure 4A, the hypothetical history of the analyzed arrays produced by CRISPRtree can be reconstructed.

From Anc_a, two arrays arose: Anc_b and Anc_f. The events in the upper clade, starting with Anc_b, are described here to illustrate how the visualization produced by CRISPRtree can be interpreted. Anc_b differs from its ancestor Anc_a by the deletion of two spacers and the acquisition of several. From Anc_b, the extant array 47 arose by the deletion of two sets of spacers and the acquisition of two spacers, and Anc_c arose through the acquisition of a single spacer. From Anc_c, the extant array 58 arose through the duplication of an existing spacer, and Anc_d arose through two deletions. The extant array 57 has no annotated events, indicating that it is identical to Anc_d, while array 56 differs from Anc_d by a single deletion. As array 57 and Anc_d are identical, array 57 can be considered to be the ancestor of array 56.

CRISPRtree uses an evolutionary model that favors spacer gain by leader-end acquisitions, and spacer loss by deletions or trailer-end loss. However, sometimes other events can be predicted. For example, in the lower clade, array 14 is hypothesized to have replaced five spacers in its middle with two new spacers. While this is annotated as an insertion event, it is also possible that the two spacers annotated as an insertion here may instead have been present in Anc_f and Anc_a, but independently lost in both Anc_b and Anc_e. These two mutually exclusive explanations for how the set of extant arrays may have arisen highlights the importance of being able to see and assess the evidence used to generate a tree topology. By clearly showing how it arrived at a given topology, CRISPRtree makes it easy for a user to decide whether the tree is a reasonable hypothesis or not.

In addition to illustrating the ancestral arrays and events, CRISPRtree can assign a measure of node support to each internal node in the tree (described in the Methods section). This node support value indicates the frequency with which a given node was seen in the trees generated during CRISPRtree's search for the most parsimonious tree. For example, the node support at Anc_e is 100%, indicating that arrays 50 and 76 were placed together in every tree inferred by CRISPRtree during its search. The node support at Anc_c, however, is 52%, indicating that in almost half of the trees inferred by CRISPRtree, a node containing only arrays 56, 57, and 58 was not present. These node support values provide an indication of how strong the phylogenetic signal is that places a set of arrays together given the evolutionary model used by CRISPRtree.

### Constrain can identify signals of HGT of CRISPR arrays

A common approach to examine the possibility of HGT is phylogenetic reconciliation. Phylogenetic reconciliation involves the comparison of the tree of an individual gene's evolution to a species tree.^34^ If the topology of a gene tree differs from the species tree, that is evidence that the gene has been horizontally transferred.

When comparing trees, it is important to assess both whether the two trees are different, and whether each tree could also explain the data underlying the other. Numerous statistical tests have been developed to test whether different tree topologies could explain the same nucleotide sequence alignment data. For example, the Shimodaira–Hasegawa (SH) test is commonly used to assess whether a set of trees are all good explanations of a sequence alignment.^35,36^

We developed Constrain to assess whether different tree topologies may be good explanations of the array relationships. Constrain reconstructs the history of a set of CRISPR arrays given a certain tree topology and indicates the events that would need to occur during the evolution of each array. While Constrain is not a statistical testing method, it can indicate whether the given topology is reasonable or whether HGT or the independent acquisition of identical spacers in different arrays would be the most parsimonious explanation of the tree topology.

To investigate whether cluster 2 arrays may have been horizontally transferred between isolates, we first determined whether the CRISPR array tree and core genome tree differ (Supplementary Fig. S1 and Fig. 4A, B). We used RF distance (which assesses the number of partitions implied by one tree but not by another) to compare the tree produced by CRISPRtree with the core genome tree.^29^ The RF distance between the two trees is 9 out of a maximum possible value of 13. The high RF distance between the two trees indicates that there is a high degree of disagreement between the inferred histories of cluster 2 CRISPR arrays and the core genome of the isolates encoding these arrays. Disagreement between the two trees is consistent with the hypothesis that HGT of CRISPR arrays has occurred.

We next assessed whether differences between the two trees are well supported by the underlying data. Bootstrap support for two of the nodes in the core genome tree is low (bootstrap support <95%—a cutoff recommended for use with UFBoot2 values).^37^ In addition, two nodes in the CRISPRtree have low support (Anc_c: 52%, Anc_d: 60%). However, major differences between the two trees are well supported. For example, Anc_f in the tree inferred by CRISPRtree places arrays 14, 50, and 76 together with 88% support. The core genome tree places the isolates encoding those three arrays (ERR431311.14, ERR431128.50, and ERR431319.76 in Fig. 4B) apart from one another with high bootstrap support. The finding that major differences between the two trees are well supported by the underlying data provides confidence that the two trees are different.

We next determined whether the topology of the CRISPRtree tree is supported by the core genome sequence alignment using the SH test implemented in IQTREE2.^21,35^ The SH test indicates that the CRISPRtree topology for cluster 2 arrays is not supported by the alignment of core genomes (*p* = 0). This indicates that the CRISPR array tree does not represent the relationships between the core genome sequences of these isolates.

Finally, we used Constrain to evaluate whether the core genome tree could explain the relationships between cluster 2 CRISPR arrays. Constrain uses the same approach as CRISPRtree to infer the ancestral state of two arrays (see the Methods section and Supplementary Methods; Supplementary Fig. S2), but is “constrain”ed to using a single fixed tree topology. After populating the tree with inferred ancestral states, Constrain then considers any spacers that were predicted to have been acquired, and checks if each spacer was acquired at two or more locations in the tree. Constrain highlights any such independent acquisitions with red boxes.

Constrain does not always identify evidence of independent acquisitions. Supplementary Figure S4 shows an example in which the tree inferred using CRISPRtree differs from the topology of a core genome SNP tree. In this example, Constrain indicates that the core genome tree would require that a spacer duplication event occurred at a different point. However, the core genome tree is equally parsimonious to the tree inferred using CRISPRtree.

When Constrain is used to assess the core genome tree of isolates encoding cluster 2 arrays, it indicates that several spacers would need to have been independently acquired multiple times (Fig. 4C). These independent acquisition events are indicated as a red box surrounding the sets of spacers that Constrain identified as also being acquired in a different array. Independent acquisition events are annotated whenever one or more spacers could have been horizontally transferred and added to an array by recombination or independently added to multiple arrays through the normal CRISPR adaptation process.

Constrain indicates that the core genome tree does not explain the relationships between cluster 2 arrays. In addition, the distribution of cluster 2 CRISPR arrays among assemblies in this data set provides evidence of HGT (Supplementary Fig. S1). Specifically, isolates encoding cluster 2 arrays are distantly related and, in some cases, more closely related to isolates that either do not encode cluster 2 arrays or have no detectable CRISPR arrays. Taken together, these observations are consistent with a hypothesis that cluster 2 arrays have been horizontally transferred between divergent isolates.

### Identifying spacer targets using Spacerblast

As cluster 2 arrays appear to have been involved in HGT events, and these isolates were sampled from a single patient population,^17^ we reasoned that arrays may spread by HGT as they provide immunity against phages or other MGEs that are present.

CRISPR systems provide immunity against MGEs through sequence-specific targeting.^2^ In addition, CRISPR targeting often requires the presence of a short sequence flanking the target called the protospacer adjacent motif (PAM).^38,39^ We developed a tool as part of CCTK, called Spacerblast, that can search for spacer matches in a sequence database and identify the presence of PAMs (Supplementary Methods). Spacerblast allows users to adjust the settings of the search as the identity of the match and requirement of the presence of a PAM may differ, depending on the research question. We used Spacerblast to assess whether the spacers in cluster 2 arrays have targets within the assemblies in our data set.

**FIG. 5.**
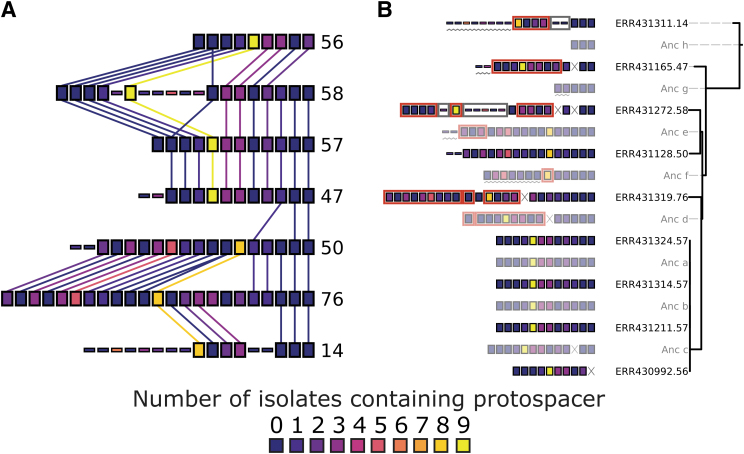
Spacerblast identifies protospacer targets of CRISPR spacers. Protospacer matches of spacers present in cluster 2 arrays were identified in assemblies using Spacerblast. Spacerblast was used with a requirement for 100% sequence identity between protospacer and spacer and the presence of a “CC” PAM 5′ of the protospacer sequence. Regions in which CRISPR arrays were identified were masked from the search using the Spacerblast “-r” option. Each spacer was assigned a color according to the number of isolates in which protospacers were identified and these color assignments were used as a custom color scheme for CRISPRdiff **(A)** and CCTK Constrain **(B)**.

We searched for spacer matches with 100% sequence identity and the presence of a type I–F “CC” PAM on the 5′ flank of the protospacer.^39^ Spacerblast identified 77 predicted protospacers with flanking PAMs (Supplementary Table S2). Each array in cluster 2 has at least one spacer with several predicted protospacers in the assemblies in this data set (Fig. 5A). In addition, the cluster 2 array spacers with the most targets are predicted by Constrain to have been independently acquired by multiple arrays (Fig. 5B). These data are consistent with the hypothesis that cluster 2 arrays have spread between isolates because they provide immunity against MGEs that infect this population.

### Assessing the performance of CRISPRtree using *in silico* evolved CRISPR arrays

As CRISPRtree uses a newly developed phylogenetic method, it is important to assess its ability to accurately infer the correct tree for a given set of arrays. However, we do not have access to a suitable data set of CRISPR arrays with a known evolutionary history to test CRISPRtree. Therefore, we developed Evolve, a tool that generates simulated CRISPR arrays with known relationships through *in silico* evolution (Supplementary Methods). Evolve is distributed as part of CCTK to allow user assessment of CRISPRtree performance.

**FIG. 6.**
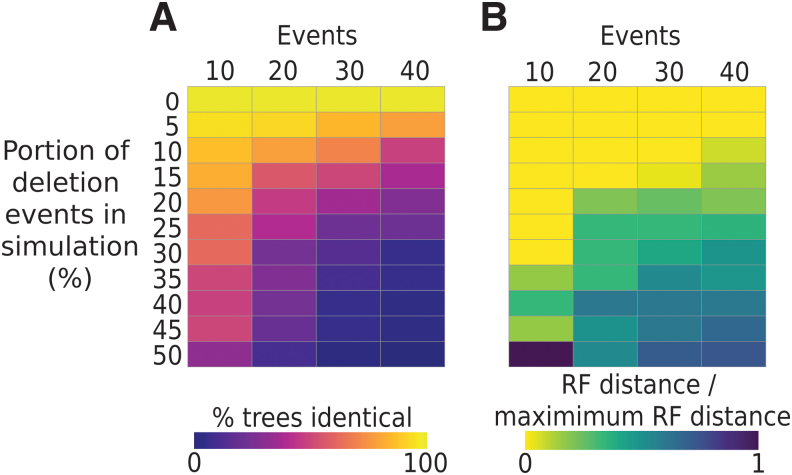
CRISPRtree can accurately recreate the true relationship between arrays when the frequency of spacer deletions is low. Simulated CRISPR arrays were produced using CCTK Evolve, and their relationships recorded in what will be referred to as the true tree topology. CRISPRtree was then used to analyze the simulated CRISPR arrays and to infer a maximum parsimony tree. The true topology of the simulated trees was compared with the topology inferred by CRISPRtree using RF distance (see the Methods section). To allow the calculation of summary statistics between multiple sets of trees, RF distance is presented as a proportion of the maximum theoretical distance for each pair of trees. Simulations were performed with a range of parameters: different proportions of spacer acquisition and deletion events (rows within each heatmap); and number of events for which each simulation was run (columns within each heatmap). For each set of parameters, 50 replicate simulations were run and two summary statistics are reported: **(A)** the proportion of trees produced by CRISPRtree that are identical to the true tree topology (RF distance of 0); **(B)** the median ratio of the observed RF distance to maximum theoretical RF distance between the CRISPRtree and true tree topology. RF, Robinson–Foulds.

We used Evolve to generate a test data set of CRISPR arrays that were generated with different evolution parameters (described in the Supplementary Methods). For each set of CRISPR arrays, Evolve stores the “true” tree describing the relationships between the simulated arrays. We then assessed the ability of CRISPRtree to infer the true tree when given only the arrays that were present at the end of each simulation (i.e., excluding any arrays that were “lost” during the *in silico* evolution process). Differences between the tree inferred by CRISPRtree and the true tree were measured using RF distance (see the Methods section).^29^ The performance of CRISPRtree for simulated data sets with different parameters is shown in Figure 6.

When CRISPRtree was used to infer a tree for sets of arrays with known relationships, its performance was impacted by the frequency of deletions in the Evolve simulations (Fig. 6). If only spacer acquisitions occurred during the simulated evolution of CRISPR arrays, then CRISPRtree produced the correct tree every time regardless of the number of events (Fig. 6A; top row). However, when spacer deletions were included in the simulated CRISPR evolution, this resulted in a reduction in the accuracy of CRISPRtree in reconstructing the relationships between arrays (Fig. 6A, B; note darker colors in lower rows).

While small numbers of deletions reduced the ability of CRISPRtree to produce the correct tree, CRISPRtree was still able to produce a tree that is similar to the true tree for most of the parameter settings tested (Fig. 6B; light colors in most rows). Furthermore, for simulations involving lower numbers of events, and therefore producing arrays that are less diverged, CRISPRtree was able to produce the correct tree more than half the time even with high frequencies of deletions (Fig. 6B).

When the tree produced by CRISPRtree differed from the true tree, it was typically because the true relationships between arrays had been obscured by deletion events, resulting in arrays for which the most parsimonious tree is not the true tree. Examples of trees produced by CRISPRtree that differ from the true tree are presented in Supplementary Figure S5. These trees show that CRISPRtree fails to identify the relationships between arrays in cases where deletions have removed evidence of those relationships.

Finally, we used the test data set generated using Evolve to assess the impact of the parsimony cost of acquisitions, deletions, insertions, and independent acquisitions events on CRISPRtree performance (Supplementary Methods; Supplementary Fig. S6). None of the tested event parsimony cost values improved CRISPRtree performance over the performance seen with default values. Instead, we found that CRISPRtree performance is robust to event cost. Specifically, CRISPRtree performance was equally good for all combinations of parsimony costs where the cost of acquisition is lowest, deletion is intermediate, and independent acquisition is highest. The parsimony cost of insertions had no impact on CRISPRtree performance in this analysis.

### Program running times

CCTK is designed to identify CRISPR arrays in a large number of assemblies, and then more closely analyze small subsets of related arrays. To illustrate approximate running times, the time taken for execution of the tools shown in this article is shown in Table 2.

**Table 2.** Example running times of CCTK tools

Tool	Running time	Comments
CCTK minced	30 s	Run on 72 assemblies
CCTK blast	51 s	Run on 72 assemblies
CRISPRdiff	1.5 s	Run on cluster 2 arrays
CRISPRtree	15 s	Run on cluster 2 arrays
Constrain	2.5 s	Run on cluster 2 arrays
Spacerblast	20 s	48 Spacers, 72 assemblies

## Discussion

In this study, we present CCTK—a toolkit for studying CRISPR arrays. CCTK includes tools to perform commonly used analyses, building on previously developed tools for the identification of CRISPR arrays (MinCED and BLASTN).^22,23^ In addition, we developed new resources for the analysis of CRISPR arrays (CRISPRdiff, CRISPRtree, Constrain, and Spacerblast).

CCTK includes two tools for the identification of CRISPR arrays: CCTK Minced and CCTK Blast. Two tools are included as the limitations and strengths of each approach means that which tool is most appropriate will depend on the needs of the user.

CCTK Minced runs MinCED and performs processing of the MinCED output.^22^ MinCED offers two major advantages: it is quick and identifies CRISPR arrays *de novo*, requiring no prior knowledge of the CRISPR types present. However, MinCED has been reported to have a high false-positive rate when identifying CRISPR arrays in some organisms and can incorrectly identify the boundaries between repeats and spacers in arrays.^40–42^ As CCTK Minced does not attempt to correct any errors in CRISPR array identification made by MinCED, CCTK Minced has the same weaknesses as MinCED. It is strongly advised that users manually check the output of CCTK Minced to distinguish true CRISPR arrays from other types of repeat regions. If needed, CCTK Minced can be rerun using manually corrected MinCED output. It is important to remember also that the quality of CRISPR array predictions depends upon the quality of the underlying assembly.

CCTK Blast uses blast to identify CRISPR arrays with known CRISPR repeat sequences.^23^ CCTK Blast does not suffer from the high false-positive rate and inaccuracy of MinCED. However, as well as requiring prior knowledge of repeat sequences, CCTK Blast is slower than CCTK Minced (Table 2).

CCTK Minced identified CRISPR arrays in the *P. aeruginosa* data set presented here accurately and with no false positives (Supplementary Table S1). When analyzing organisms in which MinCED does not perform well, manual curation of repeat sequences identified using MinCED, followed by identification of CRISPR arrays using BLAST, has been successful.^43^ By including both CCTK Minced and CCTK Blast, CCTK provides flexibility to allow a user to use either or both approaches. In addition, CCTK uses simple file formats so that outputs from other CRISPR identification tools can be analyzed.

CRISPRdiff improves upon previously developed tools for the visualization of CRISPR arrays by providing a clearer visualization of array similarities and differences. CRISPRdiff also provides easier user control of the contents and appearance of the produced plot than the existing tools and can be easily integrated into existing CRISPR-analysis pipelines.

While CRISPRdiff produces a clearer visualization, CRISPRdiff was designed to visualize small numbers of related arrays. The algorithm used to order arrays within the plot results in a long run time with large numbers of arrays.

CCTK also opens previously unexplored avenues of CRISPR research: CRISPRtree is the first tool developed for the inference of phylogenetic relationships between CRISPR arrays. CRISPRtree may be used to supplement other phylogenetic methods to further resolve relationships between highly related strains. In addition, CRISPRtree enables researchers to reconstruct the immune history of coevolving virus–host partners both in laboratory experiments and in natural systems.

Incongruence of trees is often used to detect evidence of recombination or HGT among different loci.^44^ The development of CRISPRtree to reconstruct the evolutionary histories based on nontypical parameters of CRISPR change (instead of stepwise mutation) provides the opportunity to compare CRISPR history with other loci in the chromosome to identify evidence of recombination of CRISPR alleles among strains. Constrain allows the user to assess the difference between CRISPR array relationships and other types of phylogenetic data. Previous data show that CRISPR arrays in highly variable and highly recombining regions of the chromosome are shared among strains through recombination.^45^ Constrain can improve our understanding of the role of CRISPR array HGT in the microbial pan-immune system.^46^

Spacerblast enables the prediction of protospacers in a sequence database using mismatch and PAM presence criteria. The specific search criteria that are appropriate will depend upon the research question being addressed and the biology of the organism and CRISPR system being studied.^39,47–49^ For example, if the user is interested in the current immune phenotypes of single strain, they would use a high identity and PAM criteria. If instead the user wanted to look at histories of interactions rather than current immune profiles, the user would relax these criteria. We note that short spacer blasts that allow for too many mismatches may result in spurious matches between short sequences.

In this study, we used a requirement 100% sequence identity and the presence of a PAM for a sequence to be classified as a protospacer. By using a requirement of 100% identity, we identified protospacers that are most likely to be functional immune targets of the CRISPR spacers. However, we may have missed other sequences against which the spacers would also provide functioning immunity or may have in the past. Following the acquisition of a spacer, mutations may arise in the protospacer or PAM sequence. Mutations in the protospacer sequence do not necessarily lead to immune escape, although mutations within the seed region of a protospacer may not be tolerated.^48,50^ Therefore, as our goal was to identify protospacers against which each spacer would provide immunity, a more relaxed identity cutoff could have been used.

The analysis of CRISPR arrays currently involves several steps, each of which requires distinct tools or custom code. By combining several tools into a single command-line interface, CCTK makes the analysis of CRISPR arrays easy and scalable and is a valuable new resource for the study of CRISPR systems.

## Supplementary Material

Supplemental data

Supplemental data

Supplemental data

Supplemental data

Supplemental data

Supplemental data

Supplemental data

Supplemental data

Supplemental data
