# Supplementary material for: CRISPR Comparison Toolkit: Rapid Identification, Visualization, and Analysis of CRISPR Array Diversity
Source: CRISPR J. 2023 Aug 14;6(4):386–400. doi: 10.1089/crispr.2022.0080 (PMC10457644; doi:10.1089/crispr.2022.0080)

**Figure S1. Phylogenetic relationship between isolates analyzed in this study.**

Sequence records were retrieved for the list of “clone corrected” isolates described by England et al. Assemblies were generated using Spades with the “-careful” option. Each leaf label corresponds to the European Nucleotide Archive accession number of the reads used to generate that assembly. Core genome SNPs were identified using Spine and Nucmer and aligned using a custom script (See Supplemental Methods). IQTREE2 was then used to infer a maximum likelihood tree using the core genome alignment and a model determined using the ModelFinder utiltity (model selected: TVM+F+R4). Bootstrap support was calculated using the Ultrafast Bootstrap utility which recommends a threshold of 95% support for considering a branch to be supported (Red branches not supported). The tree was visualized using iToL, midpoint rooted, and annotated according to whether any CRISPR arrays were identified (inner, black ring) and the presence of arrays belonging to the largest 7 clusters shown in Figure 2 (coloured rings; see key). Isolates encoding arrays from Cluster 2 are indicated both with a blue ring and with a blue leaf label background.


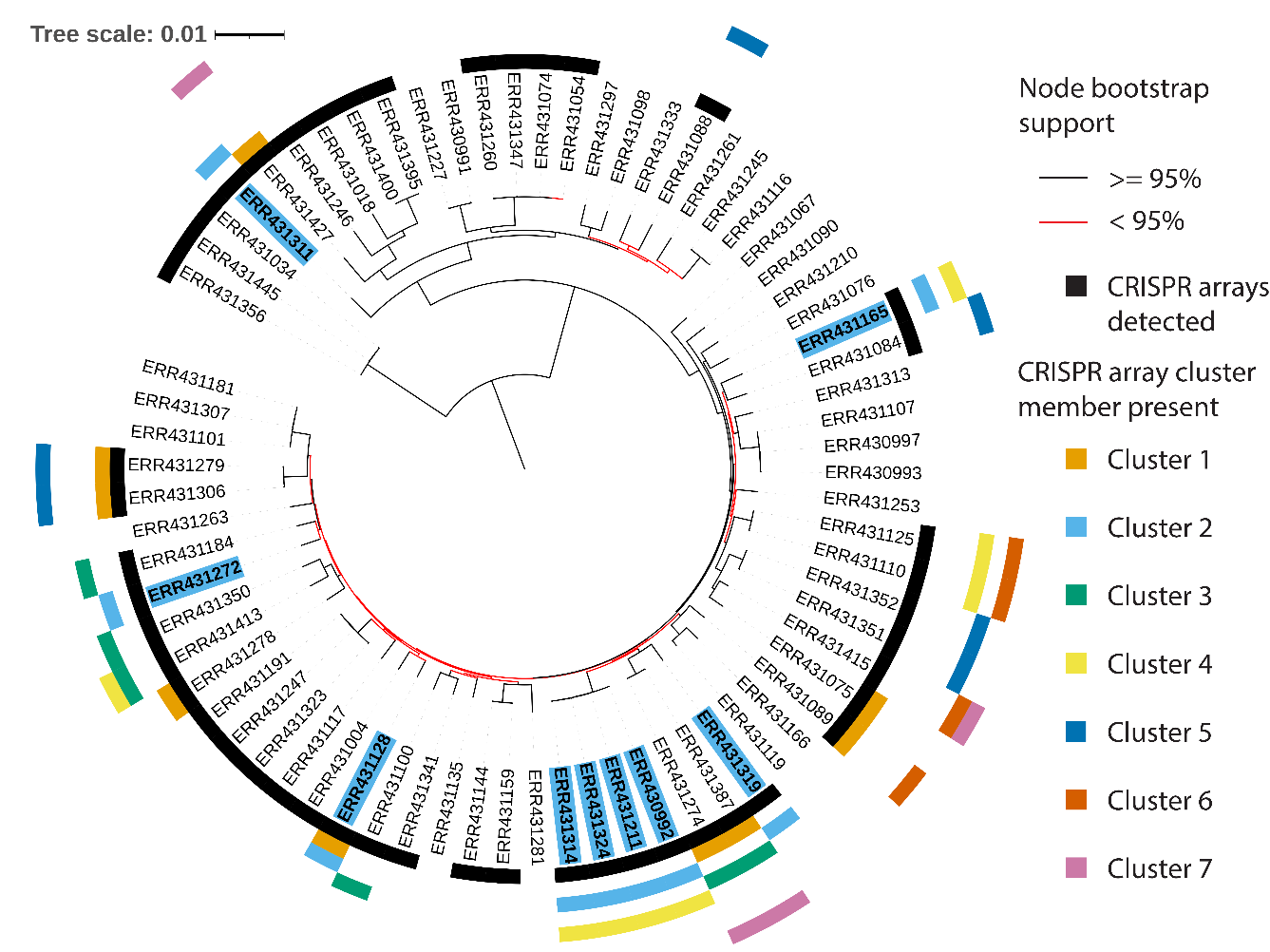

Supplement: Supplemental data [file Suppl_FigureS1.docx]
