# Supplementary material for: CRISPR Comparison Toolkit: Rapid Identification, Visualization, and Analysis of CRISPR Array Diversity
Source: CRISPR J. 2023 Aug 14;6(4):386–400. doi: 10.1089/crispr.2022.0080 (PMC10457644; doi:10.1089/crispr.2022.0080)

**Figure S2. The process used by CRISPRtree to infer a tree explaining CRISPR array relationships**

(**A**) Given a list of arrays to analyze, CRISPRtree infers a tree using the following process. (**B**) The first and second array in the list are aligned and modules of spacer in their alignment are identified (indicated using coloured bars beneath the aligned arrays). A module of spacers is a group of consecutive spacers that have the same kind of relationship between the two arrays. For example, the indel module indicated by a green bar in the middle of the aligned arrays corresponds to a set of consecutive spacers that are present in one array but not in the other. (**C**) A hypothetical ancestral array is formed. Each module is then processed to decide which spacers would be present in the ancestral array (See Supplemental Methods). (**D**) A tree is initialized with the two arrays and their ancestor. (**E**) The next array in the list is aligned to each array already present in the tree (Arrays 1, 2, and Anc_a). Modules are identified based on each alignment and the parsimony cost of each event indicated by the modules is calculated. The cost is calculated for both arrays being aligned and the greater of the two costs is taken as the score for that alignment. e.g., for the alignment of array 3 and 2, array 3 is inferred to have 3 acquired spacer, and an indel with default parsimony cost of 3+30=33. Array 2 is inferred to have 8 acquired spacers, an indel, and a trailer loss with default parsimony cost of 8+30+1=39. Therefore the parsimony cost of this alignment is 39. Array 3 when aligned with either array 1 or Anc_a has the same parsimony cost. In that case the ancestral array is preferred. (**F**) A hypothetical ancestral array is inferred for arrays 3 and Anc_a using the same process as shown in panels **B** and **C**. (**G**) Array 3 and its ancestor, Anc_b, are added to the tree. (**H**) Once all arrays in the initial list have been added to the tree, events are annotated on each array. The parsimony cost of all annotated events is used as a score for the tree. This score can be compared between trees to identify the most parsimonious tree. Different trees are produced by using a different order of arrays in the initial list.


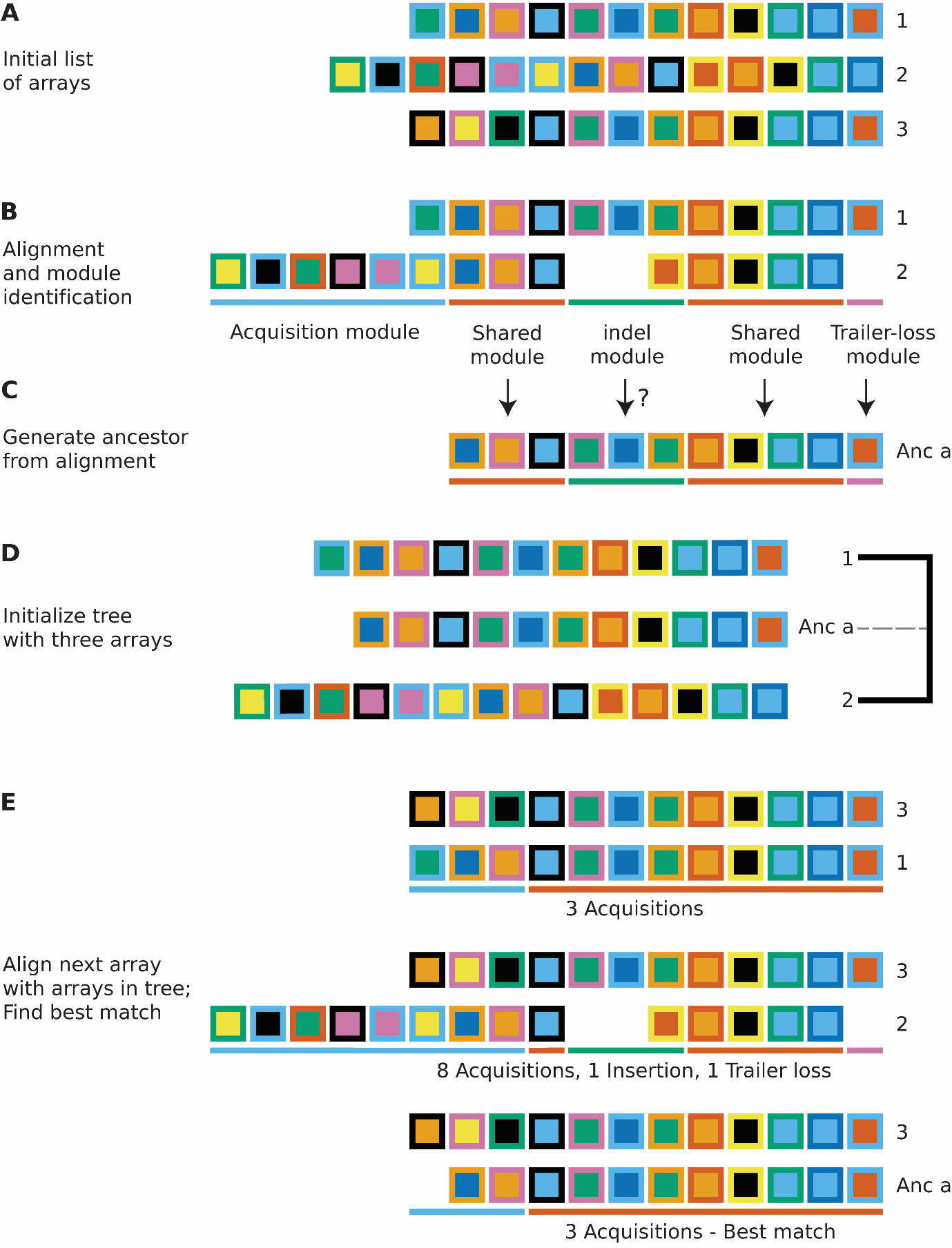

Supplement: Supplemental data [file Suppl_FigureS2.docx]
