# Supplementary material for: CRISPR Comparison Toolkit: Rapid Identification, Visualization, and Analysis of CRISPR Array Diversity
Source: CRISPR J. 2023 Aug 14;6(4):386–400. doi: 10.1089/crispr.2022.0080 (PMC10457644; doi:10.1089/crispr.2022.0080)

**Figure S3. CRISPRdiff produces a colourblind-friendly visualization while CRISPRStudio and CRISPRviz visualizations have low visual contrast.**

The arrays shown in Figure 3 are shown with the two most common forms of colourblindness simulated using Adobe Illustrator.


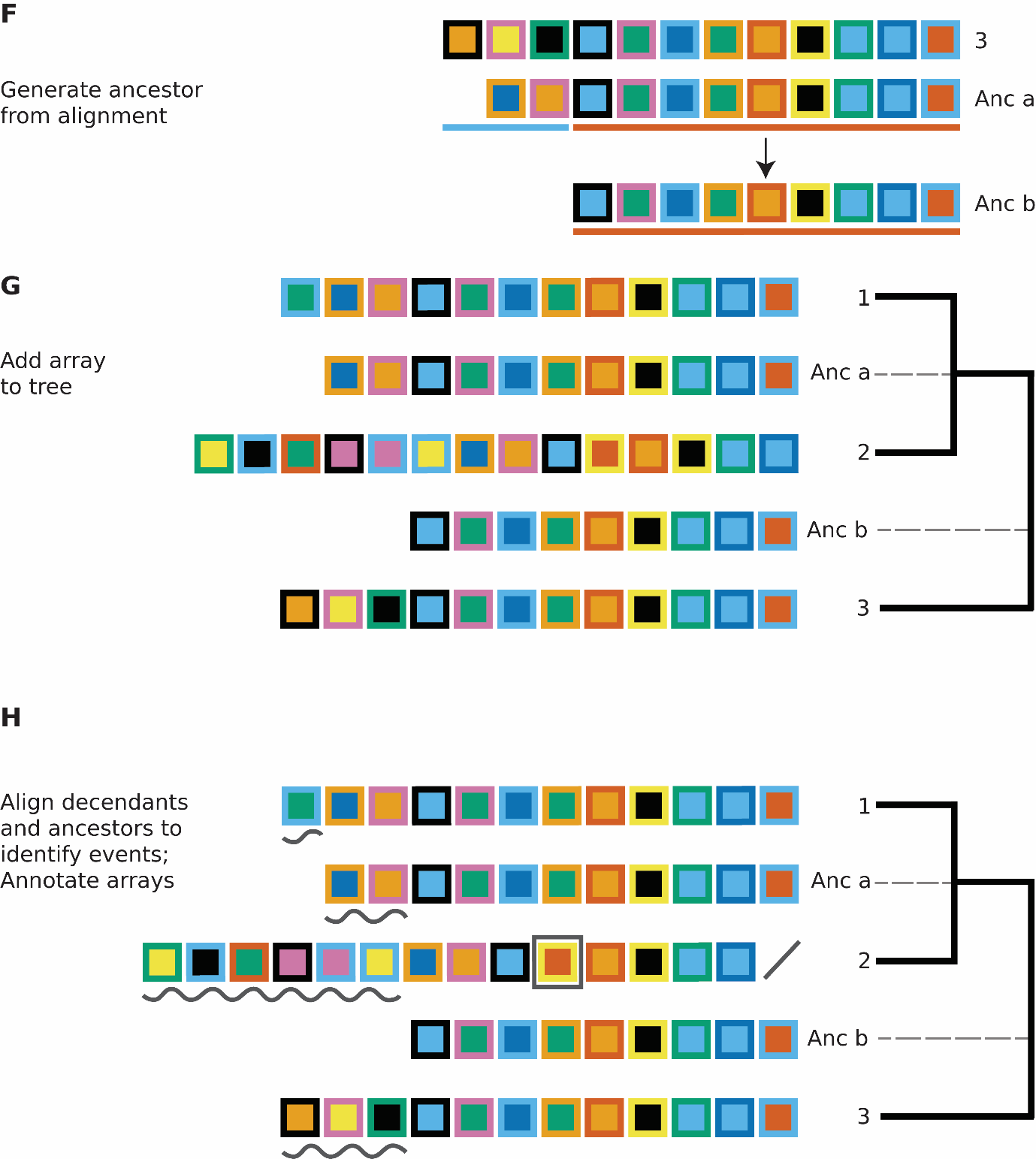

Supplement: Supplemental data [file Suppl_FigureS3.docx]
