# Supplementary material for: CRISPR Comparison Toolkit: Rapid Identification, Visualization, and Analysis of CRISPR Array Diversity
Source: CRISPR J. 2023 Aug 14;6(4):386–400. doi: 10.1089/crispr.2022.0080 (PMC10457644; doi:10.1089/crispr.2022.0080)

**Fig S4. Constrain can indicate when a tree topology is consistent with CRISPR array relationships.** Isolates encoding Cluster 7 arrays were analyzed using CCTK. (A) CRISPRdiff representation of Cluster 7 array relationships. (B) Core genome alignment was produced using Spine, Nucmer, and a custom script (Supplemental methods). A phylogenic tree was inferred using IQTREE2 (model: UNREST+FO; chosen using ModelFinder). Red branches indicate node support below 95% as calculated by the Ultrafast bootstrap tool included in IQTREE2. Leaf names are composed of the European Nucleotide Archive accession number of each isolates, a period, and the Cluster 7 array ID encoded by the isolate. (C) CRISPRtree was used to infer a tree representing Cluster 7 array relationships and the most parsimonious, best supported topology is shown. (D) Constrain was used to assess the core genome tree topology shown in panel B. Compare with Fig. 4C, note the absence of red boxes indicating no requirement for horizontal transfer of arrays or spacers.


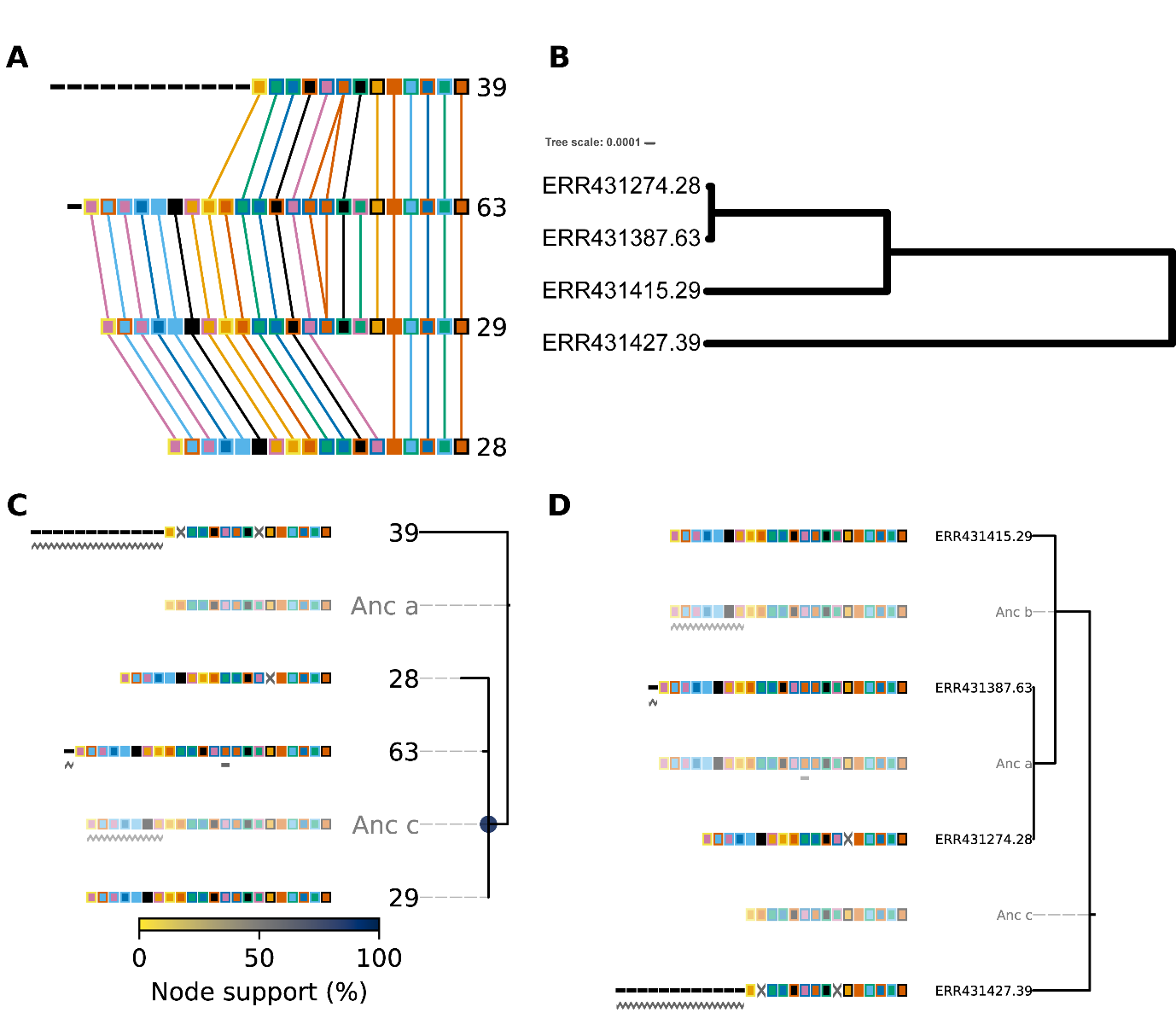

Supplement: Supplemental data [file Suppl_FigureS4.docx]
