# Supplementary material for: CRISPR Comparison Toolkit: Rapid Identification, Visualization, and Analysis of CRISPR Array Diversity
Source: CRISPR J. 2023 Aug 14;6(4):386–400. doi: 10.1089/crispr.2022.0080 (PMC10457644; doi:10.1089/crispr.2022.0080)

**Figure S5. CRISPRtree fails to produce the correct tree when arrays are degraded through deletion.**

Three examples in which CRISPRtree did not accurately identify the relationships between simulated arrays. In each panel, the tree on the left represents the true relationships of simulated arrays produced by CCTK Evolve, while the tree on the right was inferred by CRISPRtree. For each example (separate panel), arrays that have been misplaced by CRISPRtree are indicated with asterisks adjacent to the array number. Each example corresponds to a different set of simulation parameters: (**A**) 20 events, 65% acquisition, 35% deletion, 85% loss rate, (**B**) 40 events, 60% acquisition, 40% deletion, 75% loss rate, (**C**), 40 events, 70% acquisition, 30% deletion, 85% loss rate. Beneath each pair of trees, the ratio is shown of the Robinson-Foulds (RF) distance / the maximum RF distance. In the true trees, ancestral arrays are numbered according to which event number they were created by during the CCTK Evolve simulation.


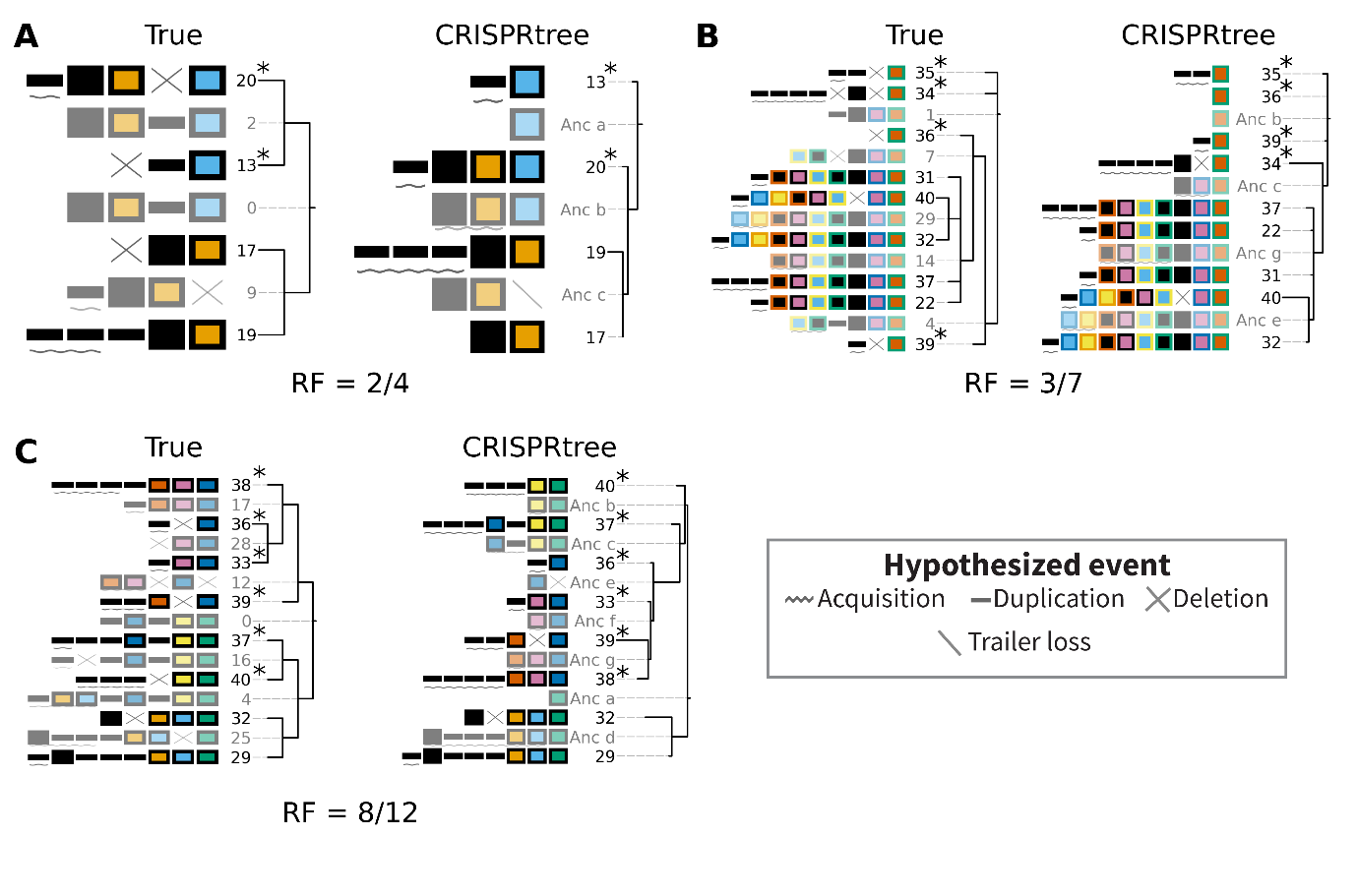

Supplement: Supplemental data [file Suppl_FigureS5.docx]
