# Supplementary material for: CRISPR Comparison Toolkit: Rapid Identification, Visualization, and Analysis of CRISPR Array Diversity
Source: CRISPR J. 2023 Aug 14;6(4):386–400. doi: 10.1089/crispr.2022.0080 (PMC10457644; doi:10.1089/crispr.2022.0080)

**Figure S6. Impact of event parsimony cost on accuracy of CRISPRtree in reconstructing true tree topology.** (**A-D**) Relative performance of CRISPRtree assessed for simulated data with different loss rates: (**A**) 75%, (**B**) 80%, (**C**) 85%, (**D**) 90%. (**E**) Relative performance of CRISPRtree using an acquisition parsimony cost of 10 when analyzing data generated with a loss rate of 75% (compare to panel **A** in which loss rate is the 75% and acquisition cost is 1). (**F**) The mean value of each heatmap shown in panels **A-E** is summarized as a single value. Shown is the impact of the parsimony cost of deletions (columns of each heatmap) and independent acquisitions (rows of each heatmap) on CRISPRtree performance. Only heatmaps for insertion cost of 30 were summarized here.


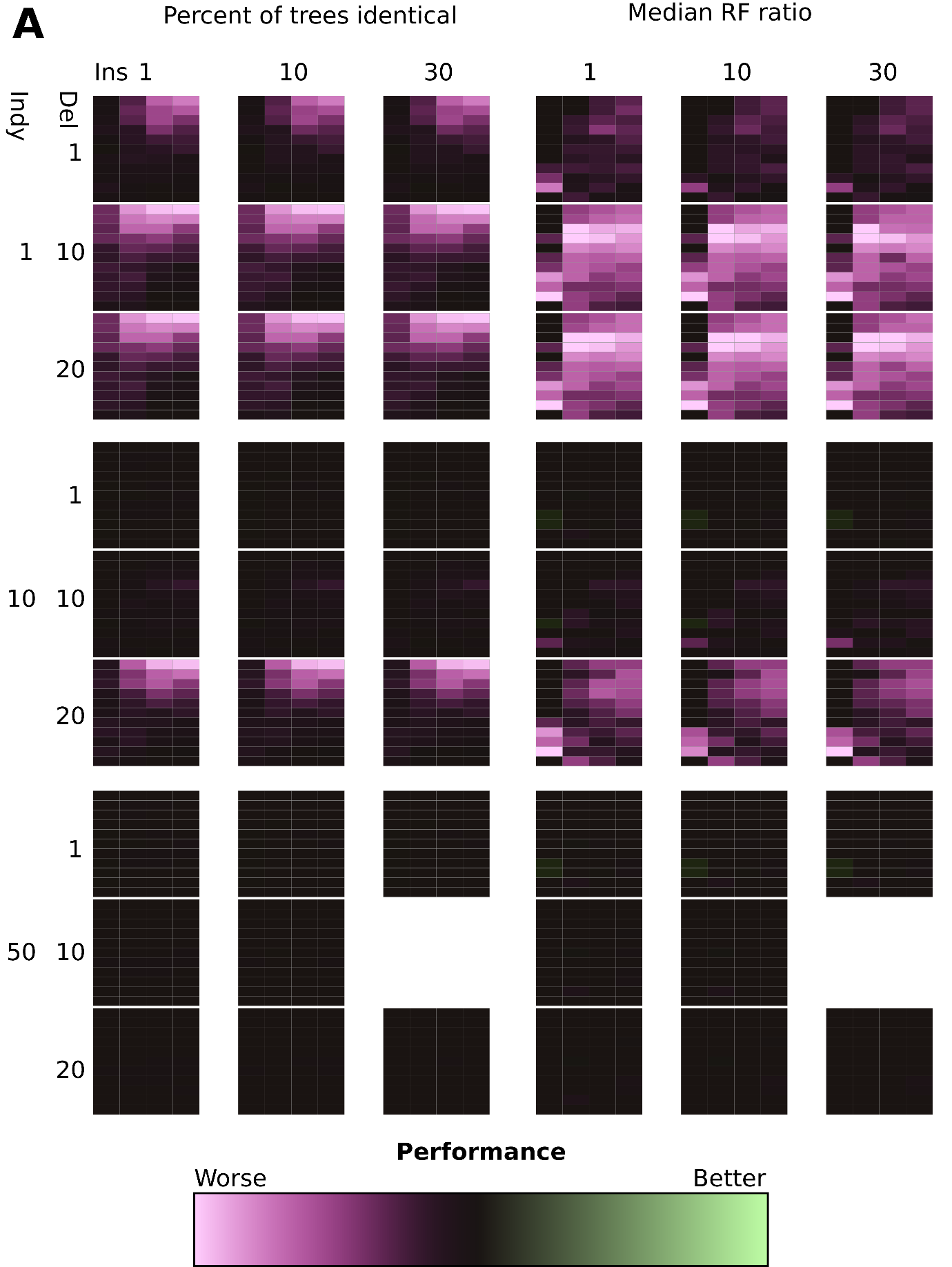

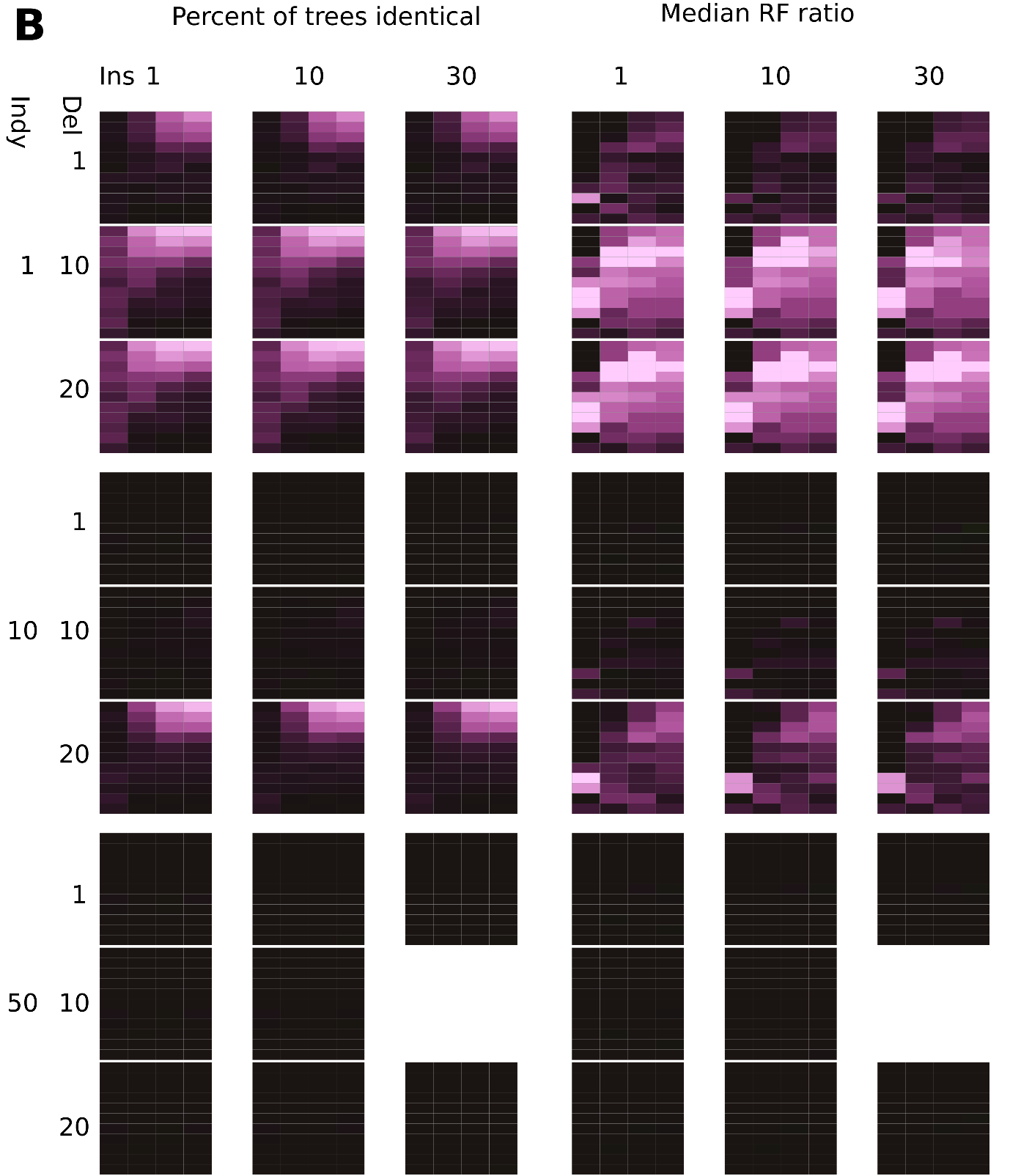

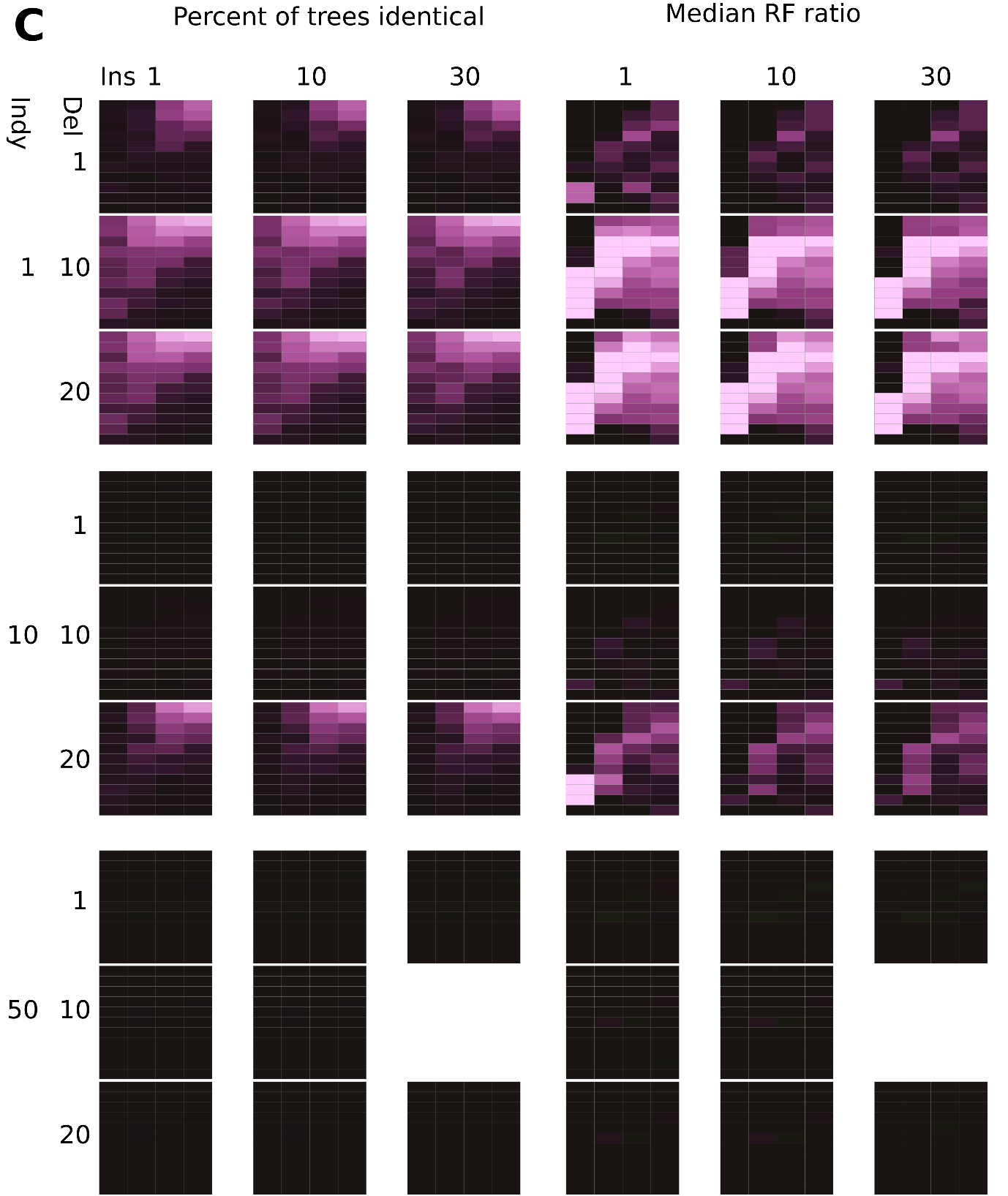

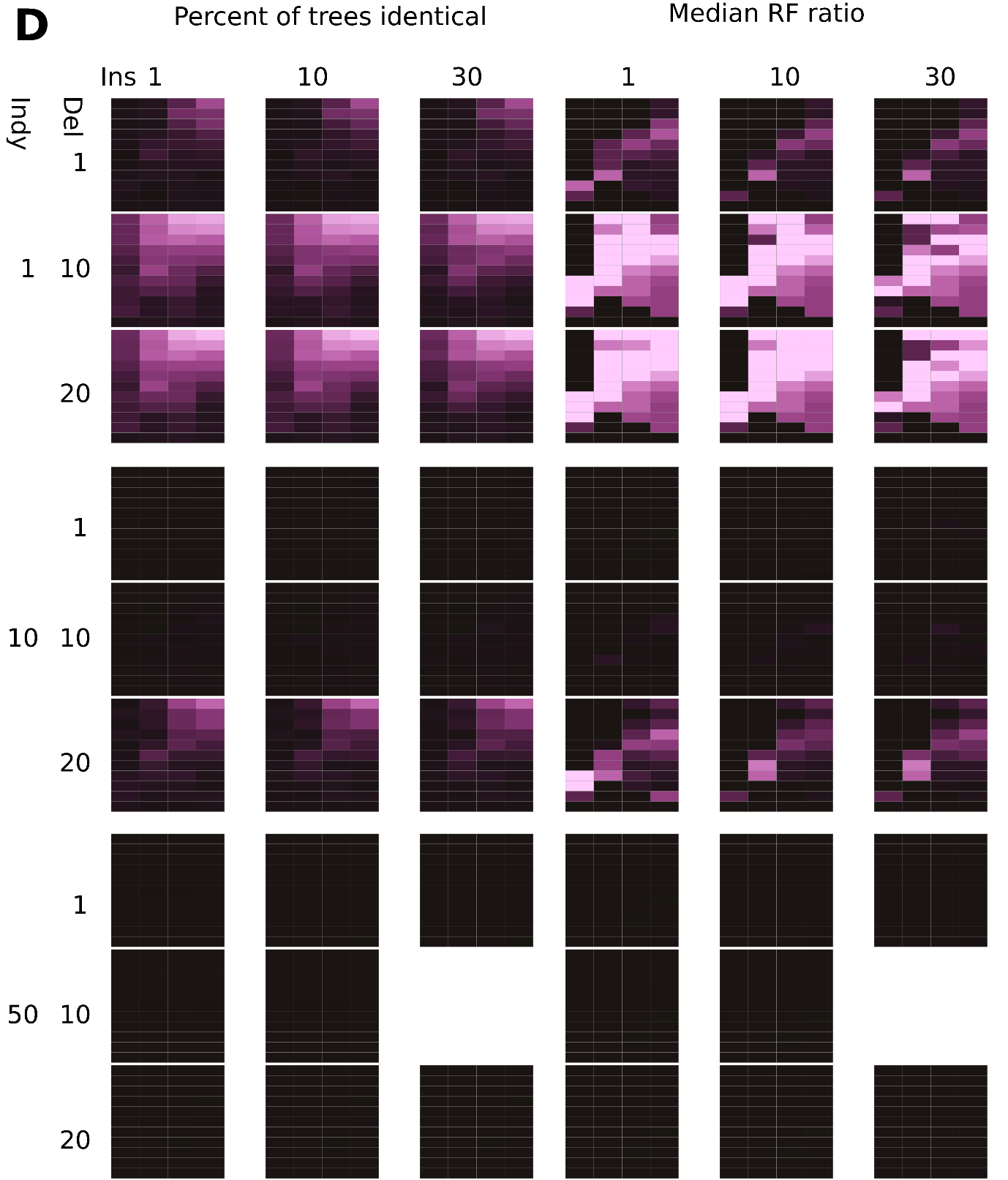

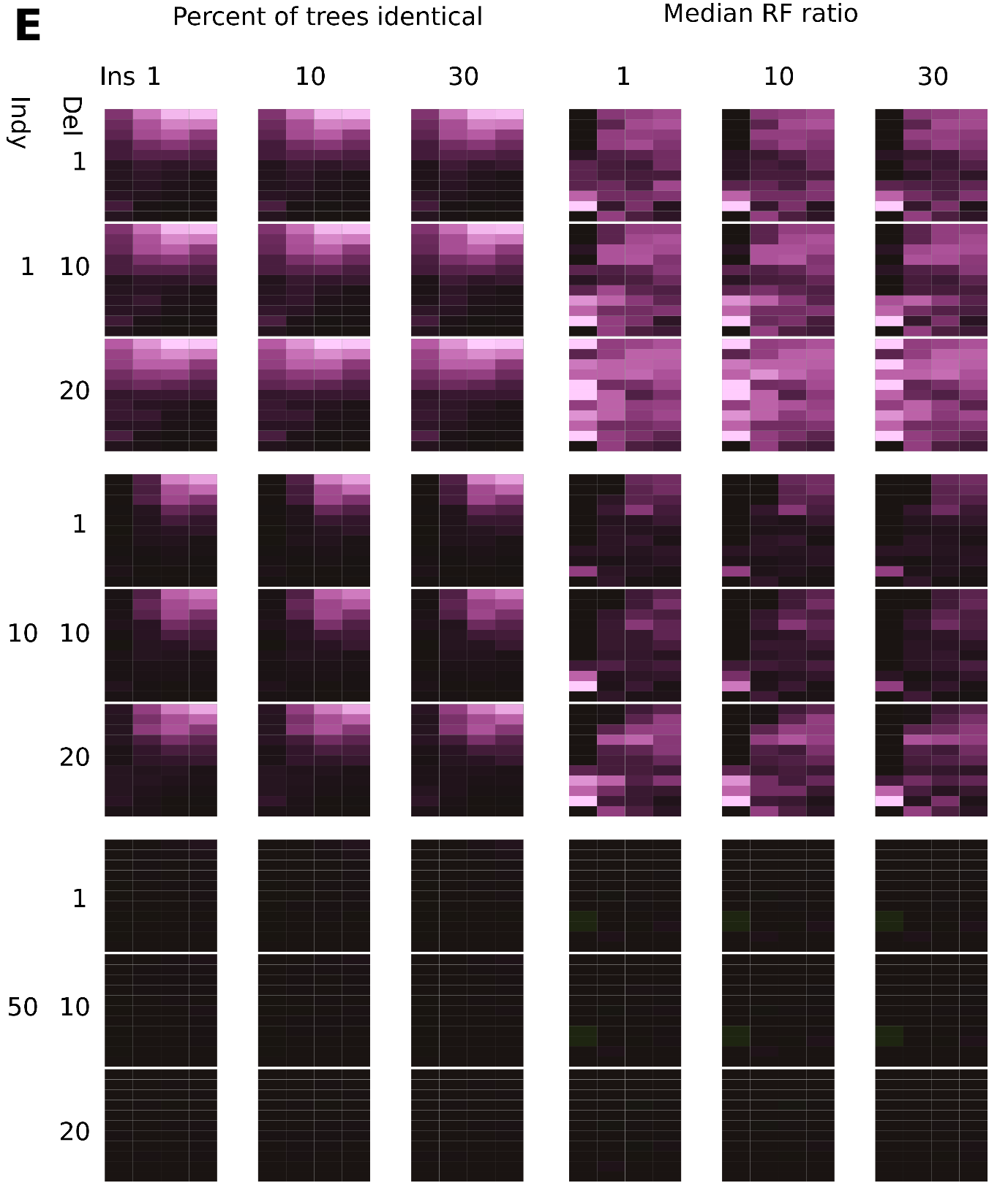

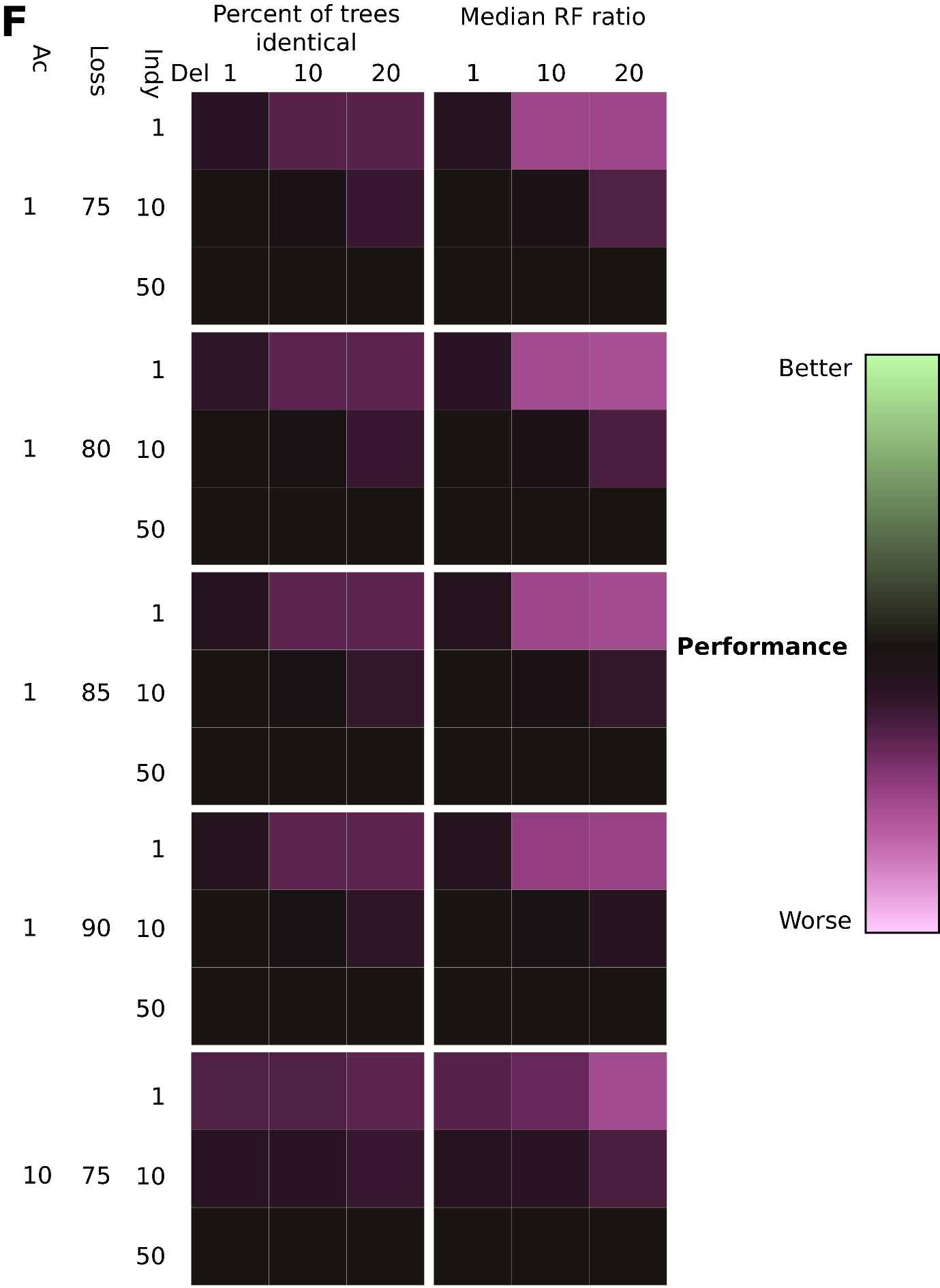

Supplement: Supplemental data [file Suppl_FigureS6.docx]
